# miRNA-1 promotes acute myeloid leukemia cell pathogenesis through metabolic regulation

**DOI:** 10.3389/fgene.2023.1192799

**Published:** 2023-05-09

**Authors:** Arevik Ghazaryan, Jared A. Wallace, William W. Tang, Cindy Barba, Soh-Hyun Lee, Kaylyn M. Bauer, Morgan C. Nelson, Carissa N. Kim, Chris Stubben, Warren P. Voth, Dinesh S. Rao, Ryan M. O’Connell

**Affiliations:** ^1^ Department of Pathology, Division of Microbiology and Immunology, University of Utah, Salt Lake City, UT, United States; ^2^ Huntsman Cancer Institute, University of Utah, Salt Lake City, UT, United States; ^3^ Department of Pathology and Laboratory Medicine, University of California Los Angeles, Los Angeles, CA, United States

**Keywords:** microRNA-1, acute myeloid leukemia, prognostic biomarker, OxPhos, hematological malignancies

## Abstract

Acute myeloid leukemia (AML) is a heterogeneous and deadly disease characterized by uncontrolled expansion of malignant blasts. Altered metabolism and dysregulated microRNA (miRNA) expression profiles are both characteristic of AML. However, there is a paucity of studies exploring how changes in the metabolic state of the leukemic cells regulate miRNA expression leading to altered cellular behavior. Here, we blocked pyruvate entry into mitochondria by deleting the Mitochondria Pyruvate Carrier (MPC1) gene in human AML cell lines, which decreased Oxidative Phosphorylation (OXPHOS). This metabolic shift also led to increased expression of miR-1 in the human AML cell lines tested. AML patient sample datasets showed that higher miR-1 expression correlates with reduced survival. Transcriptional and metabolic profiling of miR-1 overexpressing AML cells revealed that miR-1 increased OXPHOS, along with key metabolites that fuel the TCA cycle such as glutamine and fumaric acid. Inhibition of glutaminolysis decreased OXPHOS in miR-1 overexpressing MV4-11 cells, highlighting that miR-1 promotes OXPHOS through glutaminolysis. Finally, overexpression of miR-1 in AML cells exacerbated disease in a mouse xenograft model. Together, our work expands current knowledge within the field by uncovering novel connections between AML cell metabolism and miRNA expression that facilitates disease progression. Further, our work points to miR-1 as a potential new therapeutic target that may be used to disrupt AML cell metabolism and thus pathogenesis in the clinic.

## Graphical Abstract

**Figure:**
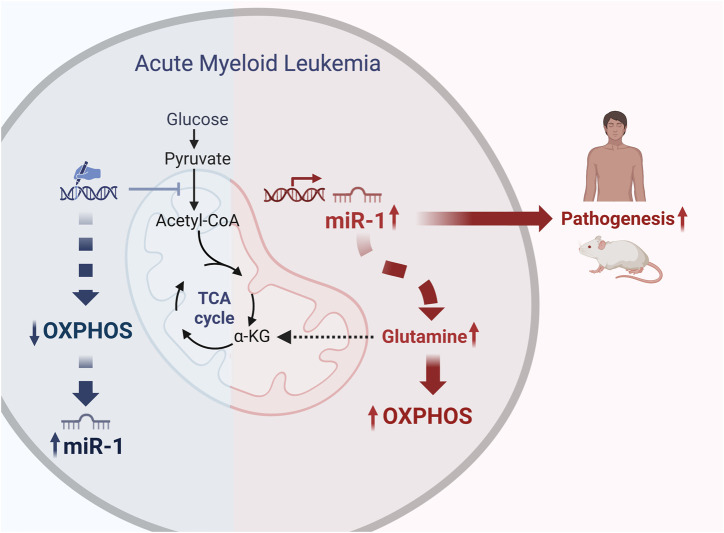
We blocked pyruvate entry into mitochondria and decreased Oxidative Phosphorylation (OXPHOS) in human AML cell lines MV4-11 and MOLM-14 by using gene editing tools. This metabolic shift led to increased expression of miR-1 in the human AML cell lines. TARGET and TCGA AML patient sample dataset analysis revealed that miR-1 is overexpressed in patients with FLT3-ITD + mutation, and independently from FLT3-ITD, high levels of miR-1 also correlates with reduced survival. Transcriptional and metabolic profiling of miR-1 overexpressing AML cells revealed that miR-1 promotes OXPHOS through glutaminolysis. Moreover, the overexpression of miR-1 in AML cells exacerbated disease in a mouse xenograft model. Together, our work expands current knowledge within the field by uncovering novel connections between AML cell metabolism and miRNA expression that facilitates disease progression. Created with BioRender.com.

## Introduction

Acute myeloid leukemia (AML) is an aggressive hematological cancer characterized by the overproduction of immature myeloid cells in the bone marrow. Despite advancements in chemotherapy, targeted small molecules, and autologous bone marrow transplantation, drug-resistant AML and rates of relapse remain challenging. This makes it necessary for a greater understanding of the molecular events which underlie AML pathogenesis and treatment responses (Ferrara and Schiffer, 2013). Relapse in patients is thought to be mediated by leukemic stem cells (LSCs), defined as a relatively rare population of cells with the ability to recapitulate bulk disease (Bonnet and Dick, 1997; Lechman et al., 2016). LSCs are characterized by distinct cell surface markers, gene expression profiles, and by a unique metabolic state characterized by an increase in oxidative phosphorylation (OXPHOS) (Lagadinou Eleni et al., 2013). This differs significantly from differentiated progeny and from normal hematopoietic stem cells. AML blasts also balance glycolysis and OXPHOS to maintain their energy demands and homeostasis (Herst et al., 2011; Chen et al., 2014; Hay, 2016). Cells obtain energy by breaking down glucose into pyruvate, followed by shuttling the pyruvate into mitochondria for OXPHOS. Mutations in metabolic genes are common in AML, such as mutations in isocitrate dehydrogenase 1 and 2 (IDH1, IDH2), key enzymes in the TCA cycle (Ragon and DiNardo, 2017; Cerchione et al., 2021; Issa and DiNardo, 2021). Using drugs to broadly target major metabolic pathways such as OXPHOS in humans is not well-tolerated despite the metabolic dependencies of AML cells. Therefore, further understanding of OXPHOS regulation in AML helps us identify key therapeutic vulnerabilities, as exemplified by B cell lymphoma 2 (BCL2) inhibition, which works in part by regulating OXPHOS (Lagadinou Eleni et al., 2013; Pollyea et al., 2018).

Mutations in FLT3 protein are the most commonly occurring mutations observed in AML patients (Daver et al., 2019). Internal tandem duplications (ITDs) within the juxtamembrane domain of FLT3 occur in 25%–30% of AMLs and result in autophosphorylation and constitutive receptor signaling leading to survival and proliferation. FLT3-ITD mutation is associated with worse survival for patients and high rate of relapse (Kottaridis et al., 2001). FLT3-ITD specific inhibitors, known as Type II Inhibitors (Sorafenib, Ponatinib and Quizatinib), have been developed and used in clinics (Daver et al., 2019), but despite the fact that they have high specificity with great potential and effectiveness, the relapse rate and resistance to these drugs remains an ongoing challenge. Interestingly, patients with FLT3-ITD mutations have a distinct metabolic profile compared to patients with FLT3-WT patients, however, the mechanistic basis for this remains largely under investigation (Stockard et al., 2018).

MicroRNAs (miRNAs) are small non-coding RNA molecules that regulate gene expression post-transcriptionally. They repress their target messenger RNA by binding to its 3′ untranslated region and reducing protein output. Regulation of cellular processes by miRNAs occurs through the modulation of a few dominant mRNA targets, and/or by simultaneously reducing the expression of a range of target genes. There are many cases in which miRNAs have been implicated in the progression of malignancies (Wallace and O'Connell, 2017). We have previously demonstrated that specific miRNAs, such as miR-155, can regulate FLT3-ITD + driven myeloproliferative disease and AML (15, 16). In addition, miRNAs can repress expression of genes that regulate metabolism (Rottiers and Näär, 2012; Hatziapostolou et al., 2013). However, there are limitations to studies exploring how changes in the metabolic state of the FLT3-ITD + leukemic cells can regulate miRNA expression and, in turn, alter cellular behavior. Here, we unravel a relatively unexplored pathway: the interplay between miRNAs, OXPHOS, and AML. As OXPHOS plays a big role in maintaining AML and LSC survival (Lagadinou Eleni et al., 2013), understanding the mechanism of specific miRNAs in this metabolic process will ultimately enable rational and effective targeting of metabolic pathways such as OXPHOS in AML cells.

In this study, we first explored how reductions in OXPHOS regulate miRNA expression in human AML cell lines. Mitochondrial Pyruvate Carrier (MPC) transporter was recently identified, elucidating the mechanism of pyruvate entry into mitochondria (Bricker et al., 2012; Herzig et al., 2012; Schell and Rutter, 2013; Schell et al., 2014). Thus, we used the deletion of *MPC1* as a tool to reduce OXPHOS in human AML MV4-11 and Molm-14 cells (Schell et al., 2017; Ramstead et al., 2020; De La Rossa et al., 2022). The deletion of *MPC1* caused a decrease in OXPHOS and specific upregulation of microRNA-1 (miR-1) in human FLT3-ITD + MV4-11 and Molm-14 AML cell lines. miR-1 is highly expressed in muscle cells and cardiomyocytes, both of which have high energy demands, where it has been linked to metabolic regulation (Zhao et al., 2007; Heidersbach et al., 2013; Wust et al., 2018). Interestingly, miR-1 is enriched in AML human LSCs (Lechman et al., 2016), and is elevated in various subtypes of pediatric AML patients (Lim et al., 2017), but its effect on AML metabolism and pathogenesis hasn’t been characterized. We provide evidence that AML patients with high miR-1 have lower survival. Moreover, the enforced overexpression of miR-1 leads to increased OXPHOS in FLT3-ITD + AML cell lines by enhancing glutaminolysis. We further demonstrate that, in a mouse xenograft model, miR-1 overexpression helps MV4-11 cells to better engraft which accelerates mortality.

## Materials and methods

### Cell culture and cell lines

MV4-11 and Molm-14 cell lines were purchased from ATCC and cultured in complete RPMI media with 10% FBS, L-Glutamine and antibiotics. The cells were grown in normoxic conditions, with 5% CO2 at 37C. MPC1 KO MV4-11 and Molm-14 cells were generated using previously described CRISPR/Cas9 (Ramstead et al., 2020) lentiCRISPRv2 construct containing a GFP selection marker and one specific short guide RNA sequence against human *MPC1*. miR-1 overexpressing (miR-1 OE) MV4-11 and Molm-14 cell lines were created by transduction with ABM pLenti-EF1α-GFP containing a MIR1-2 host gene sequence (Origene CAT#: SC400037).

### Mice

Mice used for xenograft studies were Nod SCID γc−/− (NSG), a gift from the A. Welm lab, University of Utah. All experimental procedures and husbandry were performed with the approval of the Institutional Animal Care and Use Committee and the Comparative Medicine Center of the University of Utah (IACUC protocol #- 00001557).

### Xenograft experiment

MV4-11 EV or MV4-11 miR-1 OE cells were washed twice with PBS and 1.3 × 10^6^ cells in 200ul PBS were injected into NSG mice through the lateral tail vein twice on two consecutive days. N = 11–12 sex and age matched mice were used in each group for xenograft experiments. Log-rank (Mantel-Cox) test ***p = < 0.0005 and Gehan-Breslow-Wilcoxon test ***p = < 0.0005 were used for survival plots.

### qRT-PCR

mRNA was isolated by following the manufacturer protocols using the Qiagen miRNeasy Mini kit. cDNA was generated according to the manufacturer’s protocols with qSCRIPT cDNA SuperMix (QuantaBio) for mRNA and miRNA LNA RT kit (Qiagen) for miRNA reactions.

### Flow cytometry

Splenocytes, blood and bone marrow (BM) cells were harvested from mice and depleted of red blood cells, washed, and analyzed with a BD LSR Fortessa flow cytometer (BD Biosciences). Data analysis was performed by using FlowJo software.

### Seahorse assay

miR-1 OE or EV MV4-11 and Molm-14 AML cells were plated into a 96-well Seahorse XF-96 plate. Cell densities were normalized by cell count, and the same amount of cells were plated per well in all experimental groups. miR-1 or EV MV4-11 cells were treated with 5 µM bis-2-(5-phenylacetamido-1,2,4-thiadiazol-2-yl)ethyl sulfide 3 (BPTES (TOCRIS Cat. 5301)) or DMSO and incubated for 24 h before analysis. The Seahorse XF Mito Stress test was performed using a Seahorse XF-96 analyzer by the Metabolic Phenotyping Core Facility at the University of Utah, United States. For the Mito Stress test, concentrations of the following were added into the injection ports: oligomycin A A) 1 μM, FCCP 0.5 µM B), antimycin A+ rotenone 0.5 µM C).

### Metabolomics

7.9 × 10^6^ MV4-11 miR-1 OE or MV4-11 EV cells (n = 6) were used for analysis. All GC-MS analysis was performed with an Agilent 5977b GC-MS MSD-HES fit with an Agilent 7693A automatic liquid sampler. Data was collected using MassHunter software (Agilent). Metabolites were identified and their peak area was recorded using MassHunter Quant.

### miRNA and mRNA sequencing

mRNA was isolated by following the manufacturer protocols using the Qiagen miRNeasy Mini kit (Cat.: 217084). The cell extracts were treated with Qiagen RNase free DNase by following manufacturer protocol (Cat.: 79254). Sequencer: Illumina NovaSeq 6000. Average reads for mRNA sequencing: 38.1 million. For The human GRCh38 genome and gene annotation files were downloaded from Ensembl release 102 and a reference database was created using STAR version 2.7.6a (Dobin et al., 2013). Optical duplicates were removed from the paired end FASTQ files and the trimmed reads were aligned to the reference database using STAR in two pass mode to output a BAM file sorted by coordinates. Mapped reads were assigned to annotated genes using feature Counts version 1.6.3 (Liao et al., 2014). The output files were summarized using MultiQC to check for any sample outliers (Ewels et al., 2016). Differentially expressed genes were identified using a 5% false discovery rate with DESeq2 version 1.30.0 (Love et al., 2014). The miRNA expression data is provided as Supplemental Table S1. RNA sequencing data of AML cells with MPC-1 KO or EV cells, and miR-1 OE vs EV have been deposited in the National Center for Biotechnology Information Gene Expression Omnibus under GSE220126 and GSE220134.

### TCGA analysis

The clinical and miRNA expression data from TCGA LAML (Cancer Genome Atlas Research et al., 2013) were downloaded from the Genomic Data Commons (GDC) using the TCGAbiolinks package (Colaprico et al., 2016). Survival curves were plotted using high and low expression groups (50th percentiles). The FLT3-ITD + AML miRNA expression profile was taken from our previous analysis (Wallace et al., 2017). The results shown here are in whole or part based on data generated by the TCGA Research Network (http://cancergenome.nih.gov/). The content is solely the responsibility of the authors and does not necessarily represent the official views of the National Cancer Institute or the NIH. The sample and miR-1 expression data are provided in Supplemental Table S2.

### TARGET analysis

The clinical and miRNA expression data from TARGET-AML were downloaded from the Genomic Data Commons (GDC) using the TCGAbiolinks package. The raw counts from 60 patients with IDT status were analyzed using DESeq2 version 1.30.0. Differentially expressed miRNAs in IDT positive vs. IDT negative were identified using a 5% false discovery rate while controlling for any sex-related effects.

### Luciferase assay

HEK 293T cells were transfected with plasmid containing 5 target binding sites for miR-1, downstream of a PGK promoter driven Nano-Luc Luciferase reporter, along with a plasmid containing PGK promoter driving Photinis (FF) Luciferase as a reference for normalization. Empty vector or miR-1 overexpressing vector were transfected along with the reporters to validate miR-1 regulation of the reporter. Promega N1551Nano-Glo^®^ Dual-Luciferase^®^ Reporter Assay System kit was used for to generate luminescence, which was detected and quantified using a Biotek Synergy HT multi-well plate spectrophotometer.

### Statistical analysis

Statistical analysis and graphing were done in Graphpad Prism 9.0 software unless otherwise noted. Significance *p* values were determined by using an unpaired Student t test unless otherwise noted. Quantitative data are displayed as mean ± the standard error of the mean (SEM). *p* values are shown as indicated: *p ≤ .05; **p ≤ .01; ***p ≤ .001; ****p ≤ .0001; and not significant (ns) *p* > .05.

## Results

### Deletion of *MPC1* in human AML cell lines decreases OXPHOS and increases miR-1 expression

**FIGURE 1:**
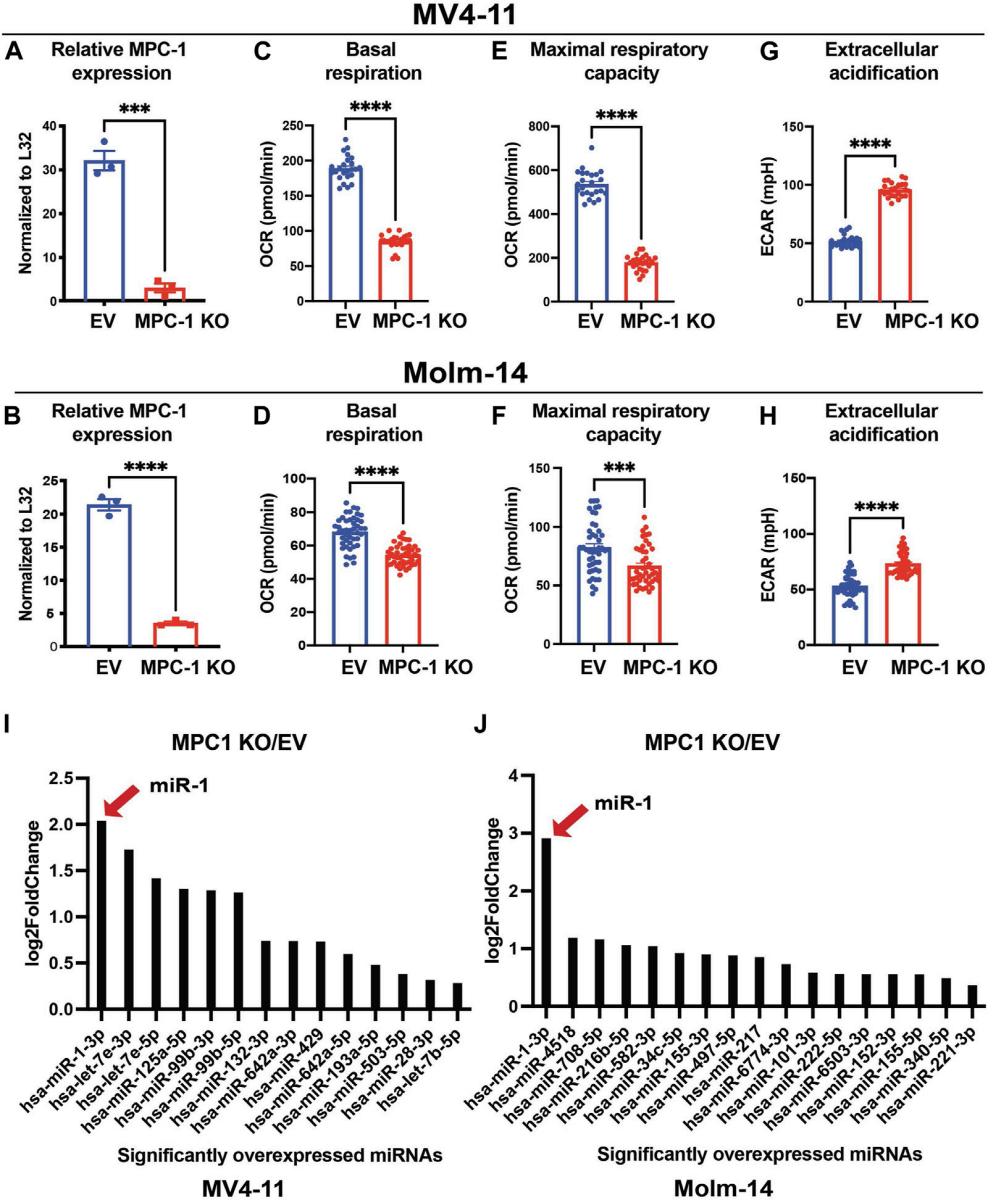
Deletion of the *MPC1* gene in human AML cell lines decreases OXPHOS and increases miR-1 expression. qPCR confirmation of MPC1 gene deletion by CRISPR/Cas9 construct in MV4-11 **(A)** and Molm-14 cells **(B)**. Mitochondrial stress test (MST) by Seahorse assay measuring basal respiration (OCR) and maximal respiratory capacity of MV4-11 WT vs MPC1 KO cells **(C, E)** and Molm-14 WT vs MPC1 KO cells **(D, F)**. Extracellular acidification (ECAR) measurements by MST Seahorse assay of MV4-11 WT vs MPC1 KO cells **(G)** and Molm-14 WT vs MPC1 KO cells **(H)**. Significantly altered miRNA expression of MV4-11 MPC1 KO vs WT cells **(I)** and Molm-14 MPC1 KO vs WT measured by whole genome miRNA sequencing **(J)** (n = 3). Unpaired two-tailed *t*-test. ***p = <0.0005, ****p = <0.0001.

To study how pyruvate-mediated OXPHOS influences miRNA profiles in AML cells, we used CRISPR/Cas9 to delete *MPC1* in human MV4-11 and Molm-14 AML cell lines. We verified our deletion by performing qPCR and confirmed a reduction in MPC1 transcripts in MV4-11 MPC1 KO (Figure 1A) and Molm-14 MPC1 KO cells (Figure 1B) compared to their nontargeting Empty Vector (EV) controls (Supplementary Figure S1A). To assess the effect of genetic deletion of MPC1 on AML cell metabolism, we used the Seahorse assay to perform metabolic analysis on MV4-11 MPC1 KO, Molm-14 MPC1 KO cells compared to EV controls. Deletion of MPC1 altered mitochondrial metabolism and OXPHOS by decreasing basal respiration and the maximal respiratory capacity of MV4-11 MPC1 KO (Figures 1C, D) and Molm-14 MPC1 KO (Figures 1E, F) cells compared to EV controls measured by Seahorse assay (Supplementary Figure S1B–E). In addition, MPC1 KO MV4-11 (Figure 1G) and Molm-14 (Figure 1H) cell lines had increased extracellular acidification, which can be interpreted as an indirect indication of reduced pyruvate mediated OXPHOS and increased pyruvate conversion to lactate. To understand how these OXPHOS alterations impact miRNA expression in AML cells, we performed RNA sequencing analysis on MV4-11 MPC1 KO vs EV, and Molm-14 MPC1-KO vs EV cells. Interestingly, miRNA-1 was the only miRNA that was induced in both MV4-11 MPC1 KO (Figure 1I) and Molm-14 MPC1 KO (Figure 1J) AML cell lines compared to EV (Supplementary Figure S1F) (Supplemental Table S1). Internal tandem duplication (ITD) in FMS-like tyrosine kinase 3 (FLT3) is one of the most common mutations observed in AML patients (Ferrara and Schiffer, 2013). MV4-11 and MOLM-14 human cell lines that were used for our initial screen have FLT3-ITD + mutations. We also knocked out MPC1 in THP1 human AML cell lines which have normal FLT3 (FLT3-ITD negative), and observed no increase in miR-1 expression (Supplementary Figures S1G, H). While each cell line had other unique and interesting miRNAs that were altered, miR-1 was consistently induced in both FLT3-ITD + cell lines. Thus, we focused our study on miR-1.

### High miR-1 expression correlates with decreased AML patient survival

**FIGURE 2:**
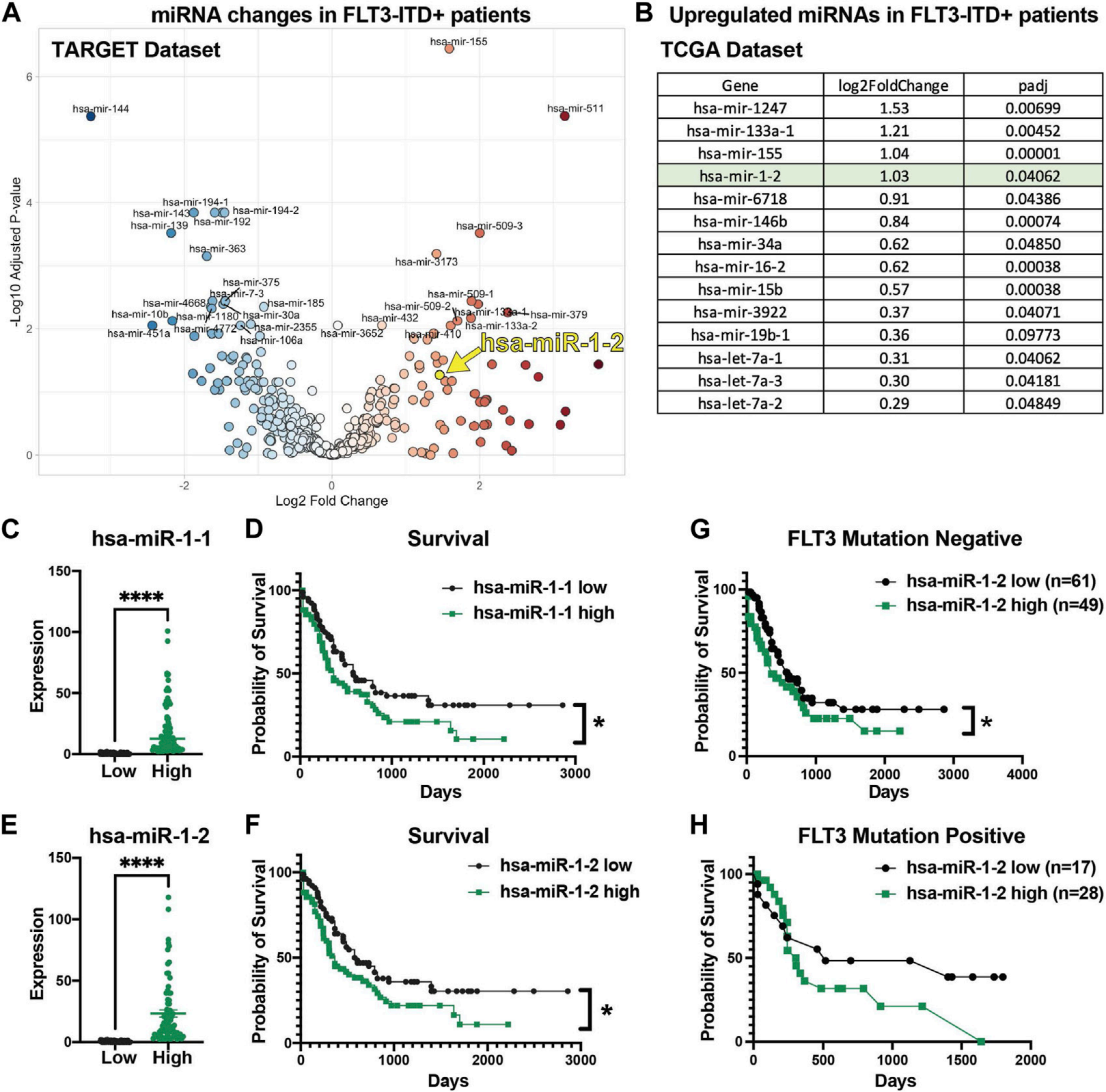
High miR-1 expression correlates with decreased AML patient survival. **(A)** Significantly upregulated and downregulated miRNAs in FLT3-ITD + AML patient samples in TARGET dataset (hsa-miR-1-2 is represented with a yellow mark). **(B)** Significantly upregulated miRNAs in FLT3-ITD + patients compared to FLT3-ITD negative patients from TCGA patient samples. Patients from TCGA dataset were divided into 2 groups on the 50th percentile high and low based on their miR-1-1 **(C)** and miR-1-2 **(E)** expression. Patients with high miR-1-1**(D)** (n = 77) or miR-1-2 **(F)** (n = 77) have worse survival compared to patients with low miR-1-1 (n = 78) or miR-1-2 (n = 78). Unpaired two-tailed *t*-test. ****p = <0.0001. Log-rank (Mantel-Cox) test was used for survival plots. *p = <0.05 Patients in TCGA dataset were divided based on their mutation status in FLT3 gene (Negative **(G)** and Positive **(H)**) and survival analysis was performed based on their miR-1-2 expression status. Gehan-Breslow-Wilcoxon test was used for survival plots. *p = < 0.05.

To extend our understanding of miR-1 in human AML and determine its clinical relevance and connection to FLT3-ITD mutation, we analyzed human TARGET miRNA sequencing datasets. Interestingly, we observed significantly increased miR-1-2 expression in FLT3-ITD patients in TARGET dataset (Figure 2A). Moreover, in our previous analysis of TCGA RNA sequencing data from FLT3-ITD + AML patient samples (Wallace et al., 2017), miR-1-2 was highly upregulated in FLT3-ITD + patients (Figure 2B), consistent with miR-1 expression being clinically relevant. We further analyzed human TCGA miRNA sequencing data and correlated miRNA expression with patient survival outcomes. Mature miR-1 can be generated from either the MIR1-1 or MIR1-2 genomic loci, so we analyzed both miR-1-1 and miR-1-2 expression in AML patients. We separated patients by the 50th percentile of miR-1-1 (Figure 2C) or miR-1-2 (Figure 2E) expression (Supplementary Table S2), with the top half of patients having high expression and the bottom half of patients having low expression. Patients with high miR-1-1 (Figure 2D) and miR-1-2 (Figure 2F), or with high mature miR-1-3p expression with high 50th percentile or 75th percentile (Supplementary Figures S2A–D) had significantly lower survival rates compared to patients with low miR-1-1 and miR-1-2, or mature miR-1-3p expression. Interestingly, we found that the worst survival occurs in miR-1-2 high patients and is not dependent on FLT3 mutation status, as patients with or without mutation in FLT3 gene have worse survival when miR-1-2 is high (Figures 2G, H). This suggests that miR-1 may be acting as an oncogene in AML patients. These results prompted us to conduct functional studies, exploring the role of miR-1 in AML cell pathogenesis.

### miR-1 overexpression in AML cell lines increases OXPHOS

**FIGURE 3:**
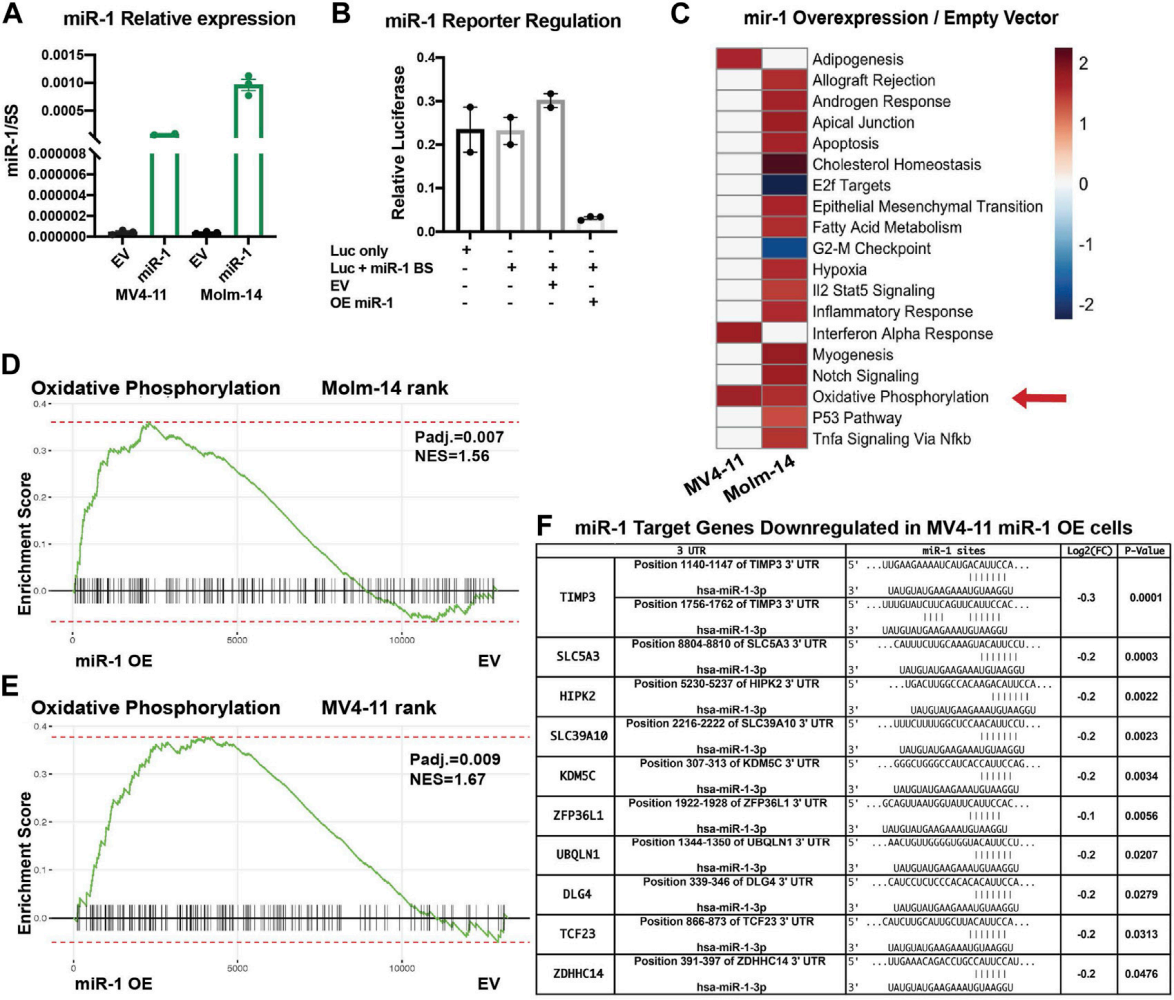
miR-1 overexpression in AML cell lines increases OXPHOS. **(A)** qPCR validation of mature miR-1 in MV4-11 miR-1 OE cells. **(B)** Luciferase activity assay measuring miR-1 binding to targeted sequence: overexpressing miR-1 (OE mir-1) with our designed vector decreases Luciferase activity, as Luciferase has miR-1 antisense binding sites (Luc + miR-1 BS). **(C)** GSEA pathway enrichment analysis of normalized enrichment scores from significantly altered gene sets when comparing miR-1 OE MV4-11 and MV4-11 EV mRNA samples using RNA-Seq. Color scale represents NES values as denoted in key. Oxidative Phosphorylation hallmark differences between MV4-11 miR-1 OE vs MV4-11 EV cells **(D)** and Molm-14 miR-1 OE vs Molm-14 EV cells **(E)**. **(F)** Table from RNA sequencing experiment of predicted miR-1 targets significantly downregulated in MV4-11 miR-1 OE cells compared to MV4-11 EV cells. Unpaired two-tailed *t*-test. *p = <0.05, ****p = <0.0001. NES, normalized enrichment score.

After determining that high miR-1 expression negatively correlates with patient survival, we wanted to investigate how high miR-1 levels influence AML cells. To accomplish this, we designed a lentiviral vector with a GFP marker to overexpress the human miR-1 hairpin in MV4-11 cells and Molm-14 cells, enabling a gain of function approach (Supplementary Figure S3A). We confirmed that our vector encodes a mature miR-1 by performing qPCR analysis (Figure 3A). miR-1 mediated suppression of targets was confirmed using reporter assays with a miR-1 anti-sense target sequence fused to the 3’ UTR of luciferase (Figure 3B) (Supplementary Figure S3B). To explore the impact of miR-1 on gene expression in AML cells, we performed RNA-Seq analyses on MV4-11 and Molm-14 miR-1 OE and EV cells. While both cell lines were altered by miR-1 overexpression, we observed largely distinct miR-1 dependent transcriptional changes in MV4-11 vs. Molm-14 cell lines (Supplementary Figures S3C–D). However, Gene Set Enrichment Analysis (GSEA) showed that the OXPHOS hallmark pathway was the only consistently upregulated pathway in both cell lines upon miR-1 OE (Figures 3C–E). Further, we also determined which genes that were reduced by miR-1 OE were candidate direct targets of repression by mir-1. This was accomplished by using the miRNA gene target prediction tool TargetScanHuman (Agarwal et al., 2015) and results identified human miR-1 binding sites in several significantly downregulated genes in miR-1 OE MV4-11 cells (Figure 3F). These findings prompted us to explore the miR-1 regulation of OXPHOS further.

### miR-1 causes metabolic changes in AML cell lines

**FIGURE 4:**
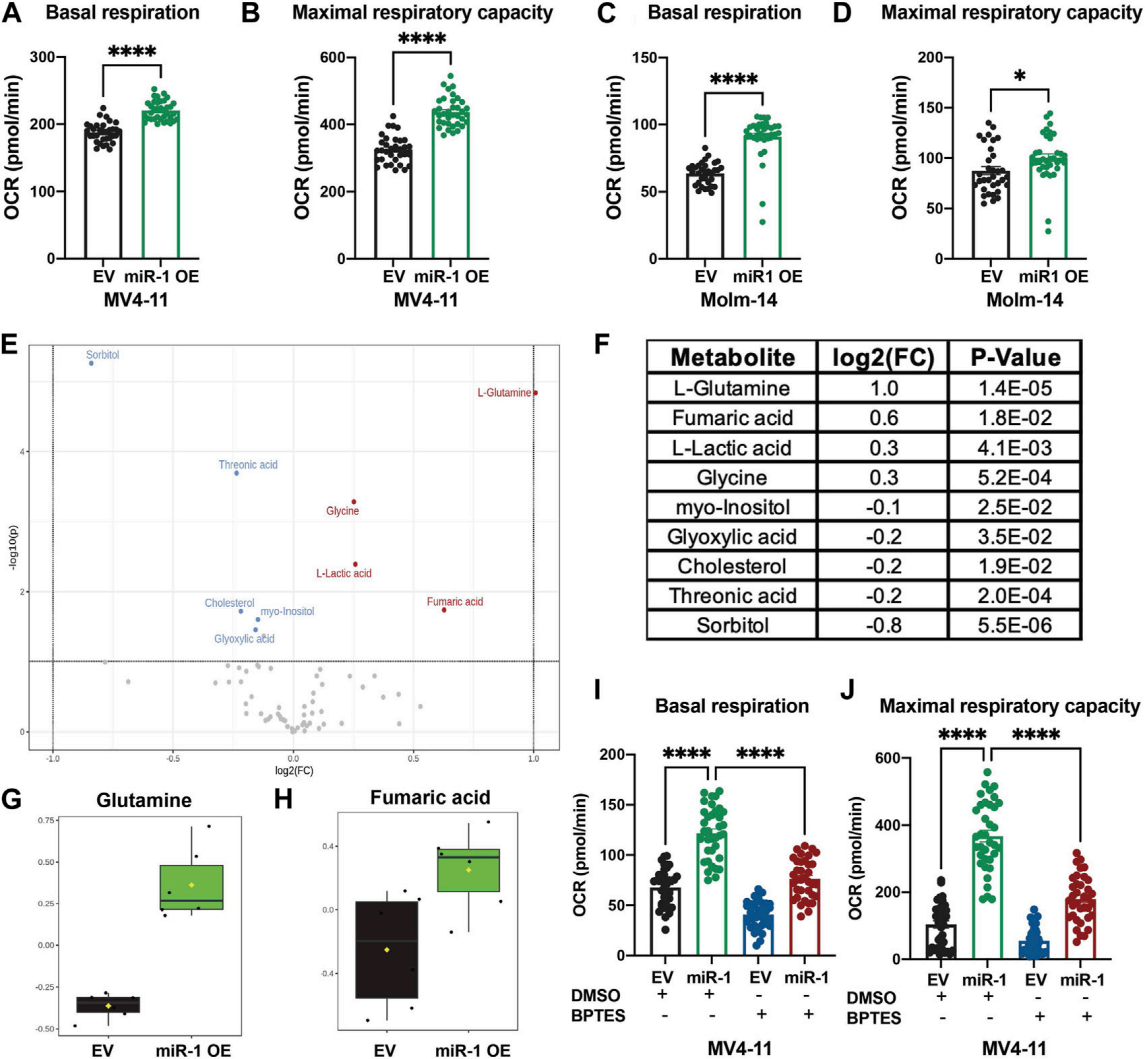
miR-1 overexpression regulates OXPHOS through glutaminolysis in MV4-11 cells. Seahorse analysis of basal respiration and maximal respiratory capacity of MV4-11 miR-1 OE vs MV4-11 EV cells **(A, B)** and Molm-14 miR-1 OE vs Molm-14 EV cells **(C, D)**. Unpaired two-tailed *t*-test. *p = <0.05, ****p = <0.0001. **(E)** Volcano plot of statistically significantly changed metabolites of MV4-11 miR-1 OE and MV4-11 EV cells with *p* = <0.05, Fold Change >1.1, raw *p*-value. **(F)** Table of statistically significantly changed metabolites with *p* = <0.05, Fold Change >1.1, raw *p*-value. Statistical analysis was performed using MetaboAnalystR. Glutamine **(G)** and Fumaric acid **(H)** levels in MV4-11 EV and MV4-11 cells. Seahorse analysis of basal respiration **(I)** and maximal respiratory capacity **(J)** of MV4-11 miR-1 OE vs MV4-11 EV cells treated with BPTES or control DMSO. Two-way ANOVA, ****p < 0.0001.

Since OXPHOS is the only pathway enriched in both MV4-11 and Molm-14 miR-1 OE cell lines, we performed metabolic phenotyping using the Seahorse assay on both cell lines. Consistent with gene expression data, the assay verified that the OE of miR-1 in MV4-11 and Molm-14 cells increased the oxygen consumption rate (OCR) during basal respiration and maximal respiratory capacity (Figures 4A–D) (Supplementary Figure S4A, B), an indicator of increased OXPHOS. We observed no significant differences in proliferation, cell death, mitochondrial mass or ROS in MV4-11 cells upon miR-1 overexpression, but they had increased Thioltracker activity with miR-1 OE (Supplementary Figures S4C–H). To extend our understanding of miR-1 regulation of OXPHOS and metabolism, we performed mass spectrometry metabolomic analyses to identify key metabolites that can be dysregulated by miR-1 OE. This identified L-Glutamine and Fumaric acid as the most upregulated metabolites in MV4-11 miR-1 OE cells (Figures 4E–H). Because glutamine metabolism through glutaminolysis is one of the pathways that supplies metabolites to the TCA cycle and OXPHOS, we hypothesized that increased OXPHOS observed in miR-1 high MV4-11 cell lines may be driven by glutaminolysis. To test that, we blocked glutaminolysis in MV4-11 OE or EV cells with GLS inhibitor bis-2-(5-phenylacetamido-1,2,4-thiadiazol-2-yl)ethyl sulfide 3 (BPTES) (Le et al., 2012; Shukla et al., 2012). BPTES reduced the basal respiration of MV4-11 miR-1 OE cells back to MV4-11 EV levels (Figure 4I), indicating that glutaminolysis may be responsible for the increased OXPHOS phenotype observed in MV4-11 miR-1 cells. In addition, BPTES decreased the maximal respiratory capacity of MV4-11 miR-1 OE cells (Figure 4J). Altogether, these results indicate that miR-1 functions to regulate OXPHOS in MV4-11 cells by promoting glutaminolysis.

### miR-1 overexpression accelerates leukemia *in vivo*

**FIGURE 5:**
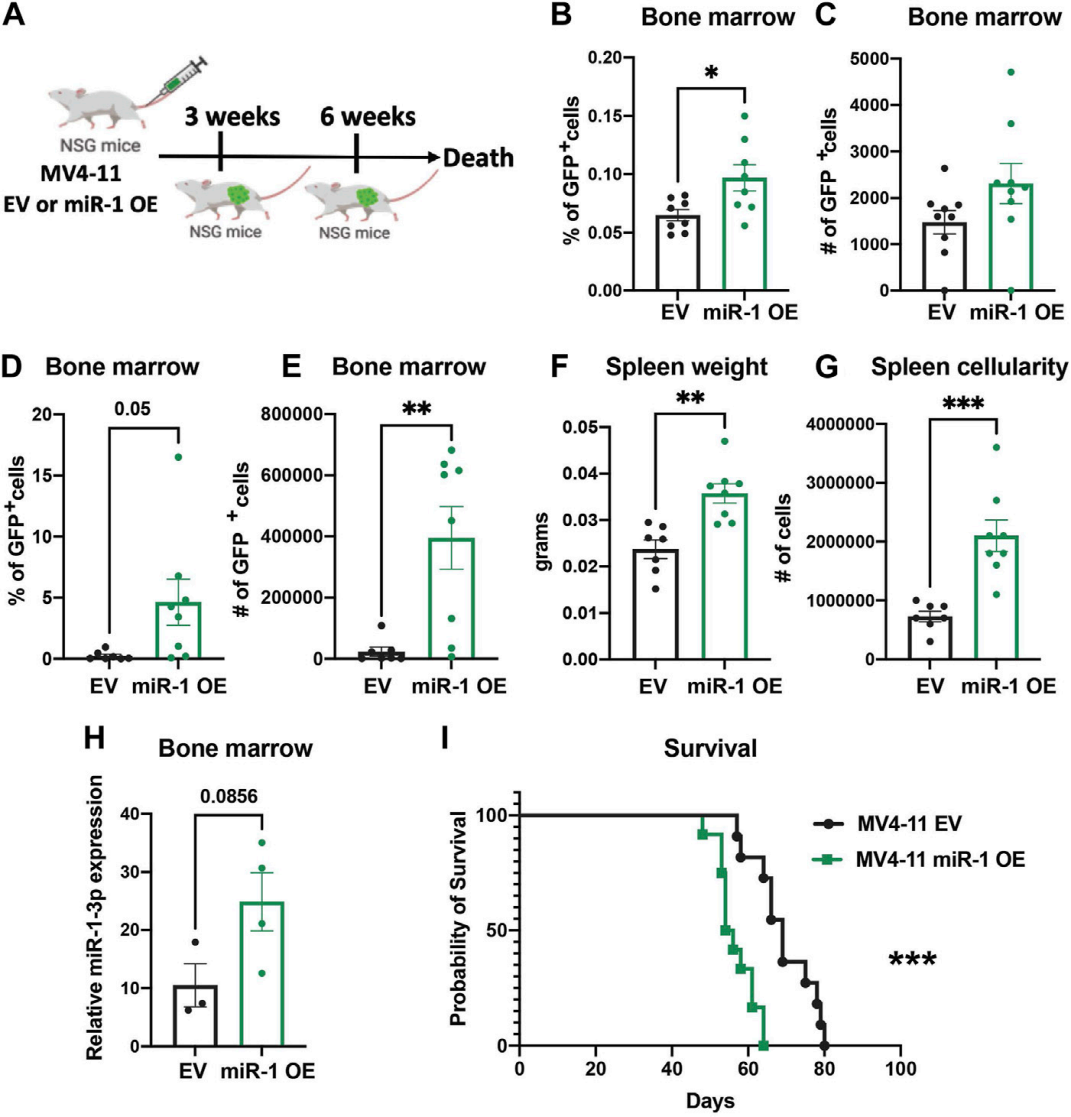
miR-1 overexpression accelerates leukemia *in vivo*. **(A)** Schematic of MV4-11 miR-1 OE and MV4-11 EV cell injections to NSG mice and the timeline of mouse bone marrow collection and analysis. Flow analysis of GFP + MV4-11 cell percentage **(B)** and total GFP + MV4-11 cell number count **(C)** of NSG mice bone marrow 3 weeks post MV4-11 miR-1 OE or MV4-11 EV cell injections. Flow analysis of GFP + MV4-11 cell percentage **(D)** and total GFP + MV4-11 cell number count **(E)** of NSG mice bone marrow, their spleen weight **(F)** and cellularity **(G)** 6 weeks post MV4-11 miR-1 OE or MV4-11 EV cell injections. For graphs B-G each dot represents a single mouse. **(H)** qPCR analysis of miR-1 levels in bone marrow of NSG mice 6 weeks post MV4-11 miR-1 OE or MV4-11 EV cell injections; each dot represents the combination of 2 different mouse bone marrow RNA. Unpaired two-tailed *t*-test. *p = <0.05, **p = <0.01. Survival of NSG mice that died when injected with MV4-11 miR-1 OE (n = 12 mice) or MV4-11 EV (n = 11mice) cells **(I)**. Log-rank (Mantel-Cox) test ***p = < 0.0005 and Gehan-Breslow-Wilcoxon test ***p = < 0.0005 were used for survival plots.

To examine whether miR-1 changes MV4-11 cell pathogenesis *in vivo*, we injected equal numbers of miR-1 OE MV4-11 cells or EV MV4-11 cells into Nod SCID γc−/− (NSG) immunodeficient mice and harvested mice 3 weeks and 6 weeks post injections (Figure 5A). At both early and late stages of disease, we observed increased AML cell expansion: the mice with miR-1 OE MV4-11 cells had increased percentage and total numbers of GFP + cells in bone marrow (Figures 5B–E), indicating that miR-1 OE confers a growth advantage to MV4-11 cells *in vivo*. Further, the mice with miR-1 OE MV4-11 cells had increased splenomegaly with increased spleen weights (Figure 5F) and cellularity (Figure 5G). qPCR analysis of bone marrow RNA showed increased miR-1 levels in mice receiving miR-1 OE MV4-11 cells (Figure 5H), thus confirming the presence of cells with the miR-1 lentivector in the bone marrow microenvironment throughout the disease time course. Importantly, there was also decreased survival of mice receiving miR-1 OE MV4-11 cells compared to EV controls (Figure 5I). This phenotype was specific to human derived AML cell lines, as we did not observe miR-1 OE-dependent regulation of OXPHOS or survival differences when transplanting the mouse leukemic cell line C1498 (Supplementary Figures S5A–D). Altogether, these findings indicate that higher miR-1 promotes human MV4-11 cell pathogenesis in a pre-clinical mouse xenograft model of AML.

## Discussion

FLT3-ITD + AML is one of the most aggressive commonly occurring mutations observed in AML patients with bad prognosis. Altered metabolomics and dysregulated miRNA profiles are characteristics observed in FLT3-ITD AML patients (Wallace and O'Connell, 2017; Lo Presti et al., 2021; Kreitz et al., 2019; Musharraf et al., 2016; Wang et al., 2019; Xiong et al., 2022). Numerous studies have found links between miRNAs and key genes involved in metabolism (Hatziapostolou et al., 2013) in different cancer models; however, little is known whether altered metabolism can cause dysregulation of miRNA expression in AML, or other cancer settings. AML cells are quite metabolically active, and interestingly both LSCs and mature AML cells utilize OXPHOS to meet their energy demands (Lagadinou Eleni et al., 2013). Here, we disrupted the TCA cycle in FLT3-ITD + MV4-11 and Molm-14 human AML cells by knocking out MPC1. This resulted in reduced OXPHOS in these cells, and increased acidification. We identified miRNAs that responded to these alterations in the cell. Interestingly, the evolutionarily conserved miR-1 was consistently upregulated in both FLT3-ITD + MV4-11 and Molm-14 cell lines when MPC1 was ablated, but not in the FLT3 WT THP-cell, which revealed an interesting connection between miR-1 and FLT3-ITD mutation.

miR-1 has been linked to leukemia (Gomez-Benito et al., 2010; Deng et al., 2017) and to a variety of solid tumor types by multiple studies (Khan et al., 2021). It has also been implicated in metabolic regulation in muscle cells and cardiomyocytes (Zhao et al., 2007; Heidersbach et al., 2013; Wust et al., 2018). However, its function in the regulation of metabolic pathways in myeloid malignancies, and specifically in AML, remain poorly understood. Our work indicates that miR-1 OE leads to increased OXPHOS in FLT3-ITD + MV4-11 and Molm-14 cells. Further, we demonstrate that miR-1 regulates OXPHOS via regulating glutaminolysis pathway in MV4-11 miR-1 OE cells. This suggests that the increase in miR-1 in MPC1 KO FLT3-ITD + cells is likely a compensatory mechanism to sustain OXPHOS using glutamine, an alternative fuel that can promote OXPHOS when pyruvate entry into the mitochondria is blocked. Interestingly, it has been demonstrated that glutaminolysis is a metabolic dependency for FLT3-ITD + leukemic cells, and combined targeting of glutamine and a FLT3 tyrosine kinase inhibitor represents better therapeutic potential (Gallipoli et al., 2018). Glutamine has been previously shown to control OXPHOS in AML cells, and targeted glutaminolysis inhibition synergized with BCL2 inhibitors, increasing their antileukemic effects (Jacque et al., 2015). The connection between glutaminolysis, miRNA-1 and OXPHOS might be ultimately utilized to block glutaminolysis, which serves as an OXPHOS compensatory mechanism, as new therapeutic strategies are developed in combination therapies with FLT3 tyrosine kinase inhibitors.

Clinically, we observed that high miR-1 expression correlates with poor patient survival. miR-1 is also highly expressed in FLT3-ITD + patients. As patients with FLT3-ITD + mutations are associated with less favorable outcomes, there is a possibility that miR-1 is upregulated as part of an aggressive leukemogenic gene expression program. Interestingly, in FLT3-ITD- patients, who generally have more favorable outcomes compared to FLT-ITD+, miR-1 plays a prognostic role of worse outcome. In our experimental model, we discovered miR-1 overexpression upon MPC1 deletion, which may reflect a cellular adaptation to metabolic disruption. In concordance with clinical findings, our *in vivo* data indicate that miR-1 may be key to driving a more aggressive phenotype in leukemia. This may be due to several reasons, including the recent demonstration that LSCs have increased OXPHOS. By supporting OXPHOS, miR-1 may render a cellular metabolic state that promotes LSC formation and retention. This is also of clinical relevance as LSCs are thought to be a key driver of relapse. The next logical step is to evaluate whether concurrently targeting metabolic vulnerabilities and miRNAs can further inhibit leukemogenesis, and this will be a focus of future studies. In this rapidly evolving field, the addition of a miR-1 inhibitor may allow for an even more significant response, given the interplay between OXPHOS, glutaminolysis and miR-1 that we have discovered in this study.

To begin to identify key miR-1 targets in MV4-11 cells we used RNA sequencing data to identify genes that are downregulated in miR-1 OE MV4-11 cells and have conserved miR-1 binding sites. miR-1 has multiple targets and its impact on OXPHOS might be a result of miR-1 simultaneously “buffering” the expression of a range of target genes that impact this key metabolic process. Among miR-1 targets is KDM5C, which is a Lysine-specific demethylase regulating genes involved in metabolism. KDM5C cross talks with IDH1/2 and alpha-ketoglutarate, connecting it to AML metabolism (Chang et al., 2019). In addition, the miR-1 target SLC5A3 is a myo-inositol cotransporter (Wei et al., 2022), and metabolomics analysis revealed reduced myo-inositol in MV4-11 miR-1 OE cells. This introduces the possibility that miR-1 can bind and downregulate SLC5A3, causing reduced myo-inositol levels in the cells. Whether SLC5A3 or KDM5C genes are involved in MV4-11 cell metabolism by regulating glutaminolysis remains unclear and will be investigated during future studies, along with other, non-canonical mechanisms that have recently been linked to miR-1 function (Zhang et al., 2014; Seok et al., 2020). Interestingly, miR-1 overexpression caused distinct transcriptional changes in MOLM-14 and MV4-11 cells, suggesting that the background of the cell lines may dictate how miR-1 regulates its varying targets. Post-transcriptional regulation of miR-1 has been looked at in other cell types. A recent study has demonstrated that reactive oxygen species (ROS) lead to position-specific oxidation of miR-1 and redirection of its target repression to genes involved in cardiac hypertrophy pathways (Seok et al., 2020). Alternatively, miR-1 has been shown to enter mitochondria during muscle differentiation and stimulate translation rather than the repression of mitochondrial specific transcripts (Zhang et al., 2014). This evidence highlights the complex relationship between miR-1 and mitochondrial function and activity in eukaryotic cells, which might be an alternative or additional mechanism of miR-1 regulating OXPHOS in AML cells. Future work will clarify the mechanistic basis for miR-1 in AML cells, and provide a basis for the development of novel therapeutic interventions.

## Data Availability

The datasets presented in this study can be found in online repositories. The names of the repository/repositories and accession number(s) can be found in the article/Supplementary Material.
