# Supplementary material for: miRNA-1 promotes acute myeloid leukemia cell pathogenesis through metabolic regulation
Source: Front Genet. 2023 May 9;14:1192799. doi: 10.3389/fgene.2023.1192799 (PMC10203238; doi:10.3389/fgene.2023.1192799)
Supplement: Supplementary file 1 [file Presentation1.pptx]

## Slide 1
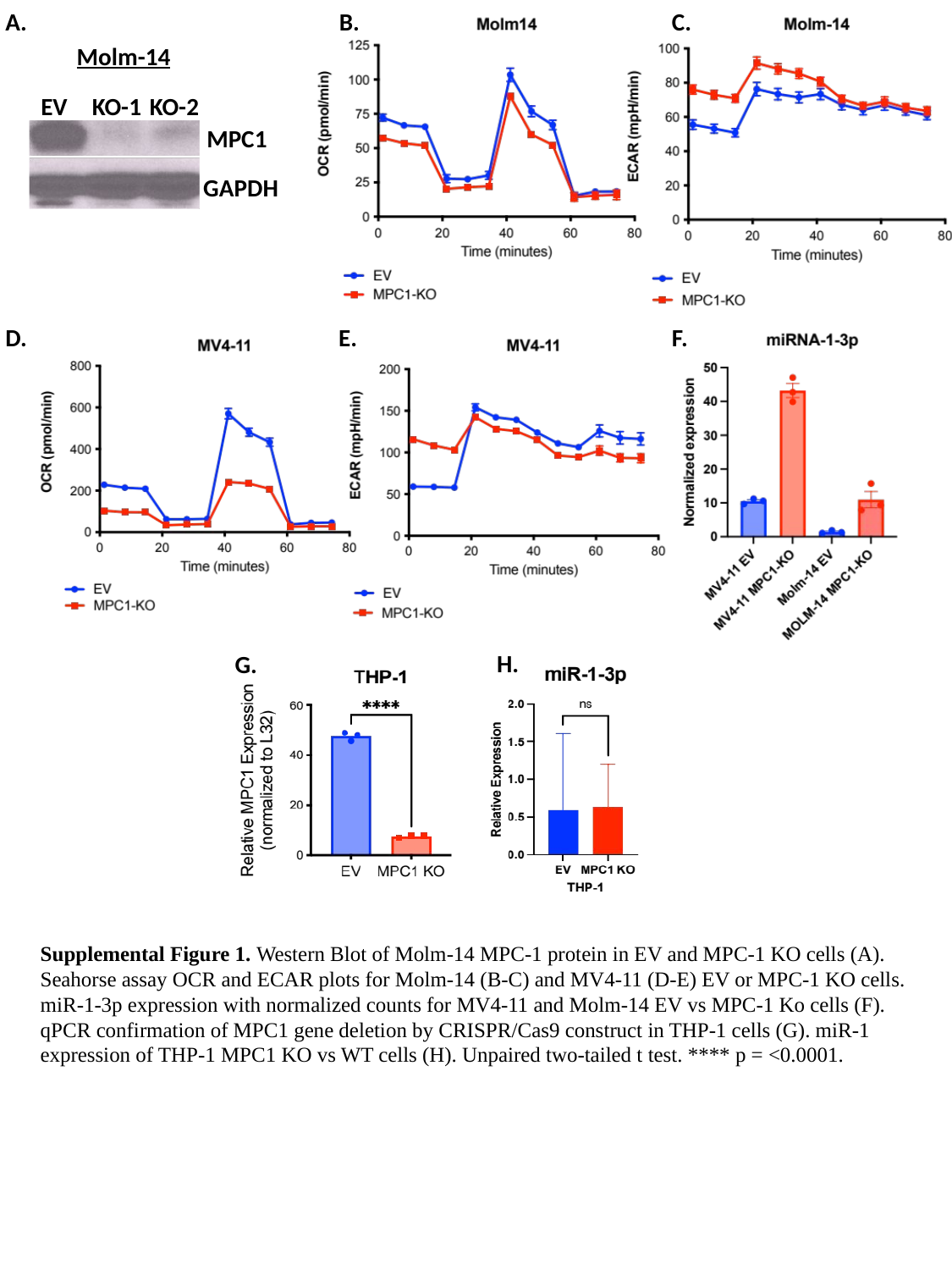

A.
B.
C.
D.
E.
F.
H.
G.
Supplemental Figure 1. Western Blot of Molm-14 MPC-1 protein in EV and MPC-1 KO cells (A). Seahorse assay OCR and ECAR plots for Molm-14 (B-C) and MV4-11 (D-E) EV or MPC-1 KO cells. miR-1-3p expression with normalized counts for MV4-11 and Molm-14 EV vs MPC-1 Ko cells (F). qPCR confirmation of MPC1 gene deletion by CRISPR/Cas9 construct in THP-1 cells (G). miR-1 expression of THP-1 MPC1 KO vs WT cells (H). Unpaired two-tailed t test. **** p = <0.0001.
Molm-14
EV
KO-1
KO-2
MPC1
GAPDH

## Slide 2
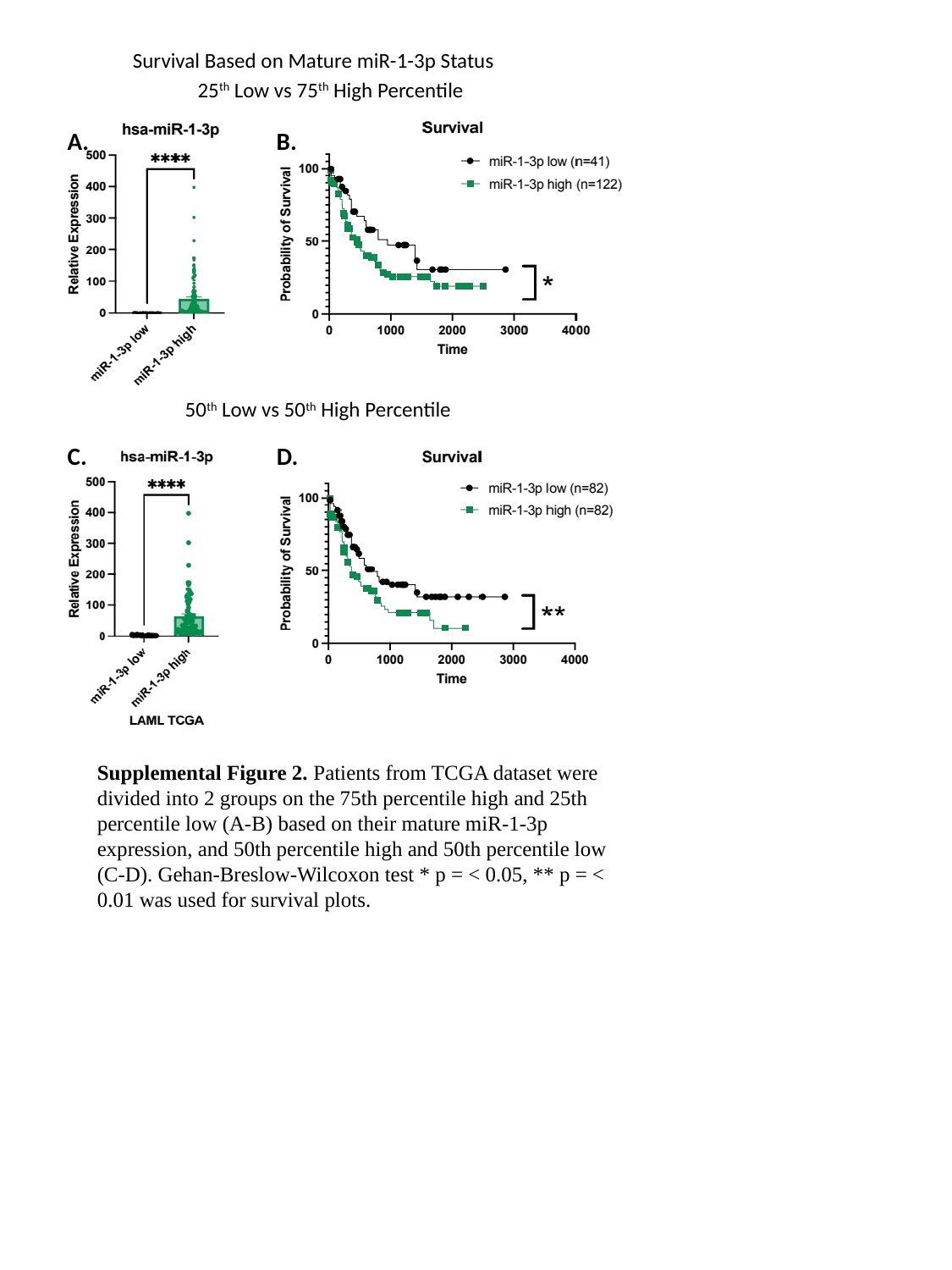

Survival Based on Mature miR-1-3p Status
25th Low vs 75th High Percentile
A.
B.
50th Low vs 50th High Percentile
C.
D.
Supplemental Figure 2. Patients from TCGA dataset were divided into 2 groups on the 75th percentile high and 25th percentile low (A-B) based on their mature miR-1-3p expression, and 50th percentile high and 50th percentile low (C-D). Gehan-Breslow-Wilcoxon test * p = < 0.05, ** p = < 0.01 was used for survival plots.

## Slide 3
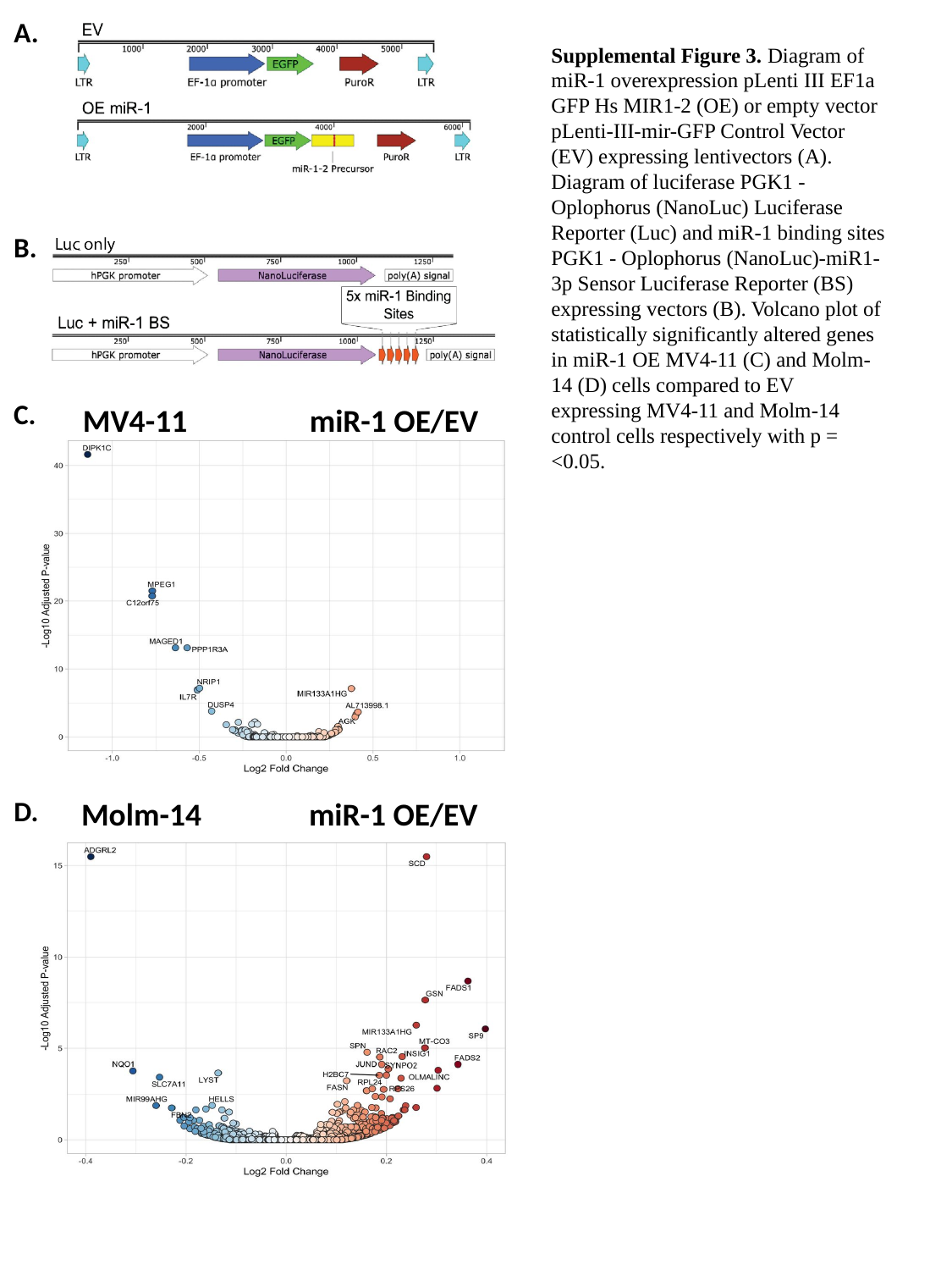

A.
B.
C.
MV4-11
miR-1 OE/EV
D.
Molm-14
miR-1 OE/EV
Supplemental Figure 3. Diagram of miR-1 overexpression pLenti III EF1a GFP Hs MIR1-2 (OE) or empty vector pLenti-III-mir-GFP Control Vector (EV) expressing lentivectors (A). Diagram of luciferase PGK1 - Oplophorus (NanoLuc) Luciferase Reporter (Luc) and miR-1 binding sites PGK1 - Oplophorus (NanoLuc)-miR1-3p Sensor Luciferase Reporter (BS) expressing vectors (B). Volcano plot of statistically significantly altered genes in miR-1 OE MV4-11 (C) and Molm-14 (D) cells compared to EV expressing MV4-11 and Molm-14 control cells respectively with p = <0.05.

## Slide 4
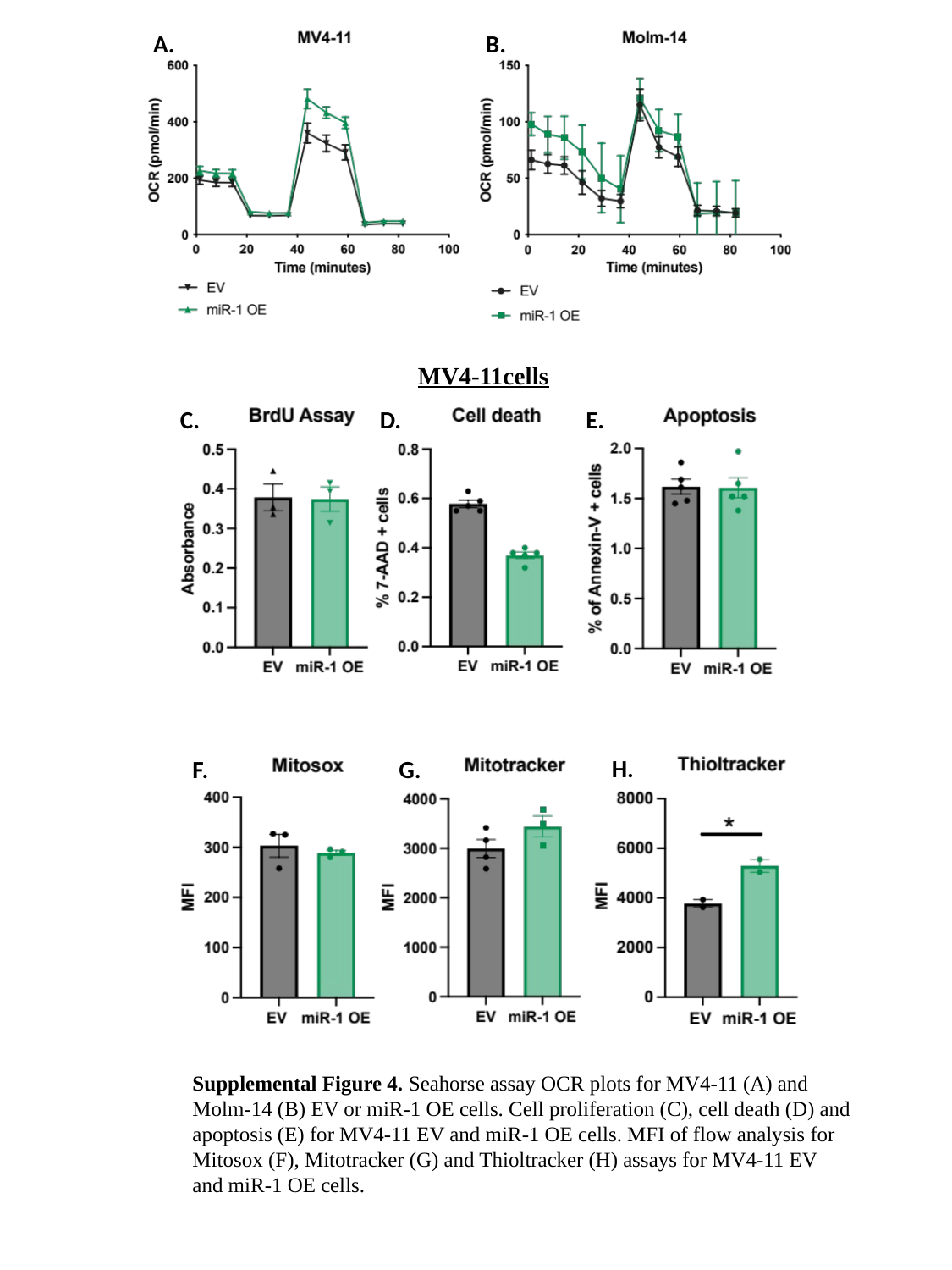

A.
B.
MV4-11cells
C.
D.
E.
H.
F.
G.
Supplemental Figure 4. Seahorse assay OCR plots for MV4-11 (A) and Molm-14 (B) EV or miR-1 OE cells. Cell proliferation (C), cell death (D) and apoptosis (E) for MV4-11 EV and miR-1 OE cells. MFI of flow analysis for Mitosox (F), Mitotracker (G) and Thioltracker (H) assays for MV4-11 EV and miR-1 OE cells.

## Slide 5
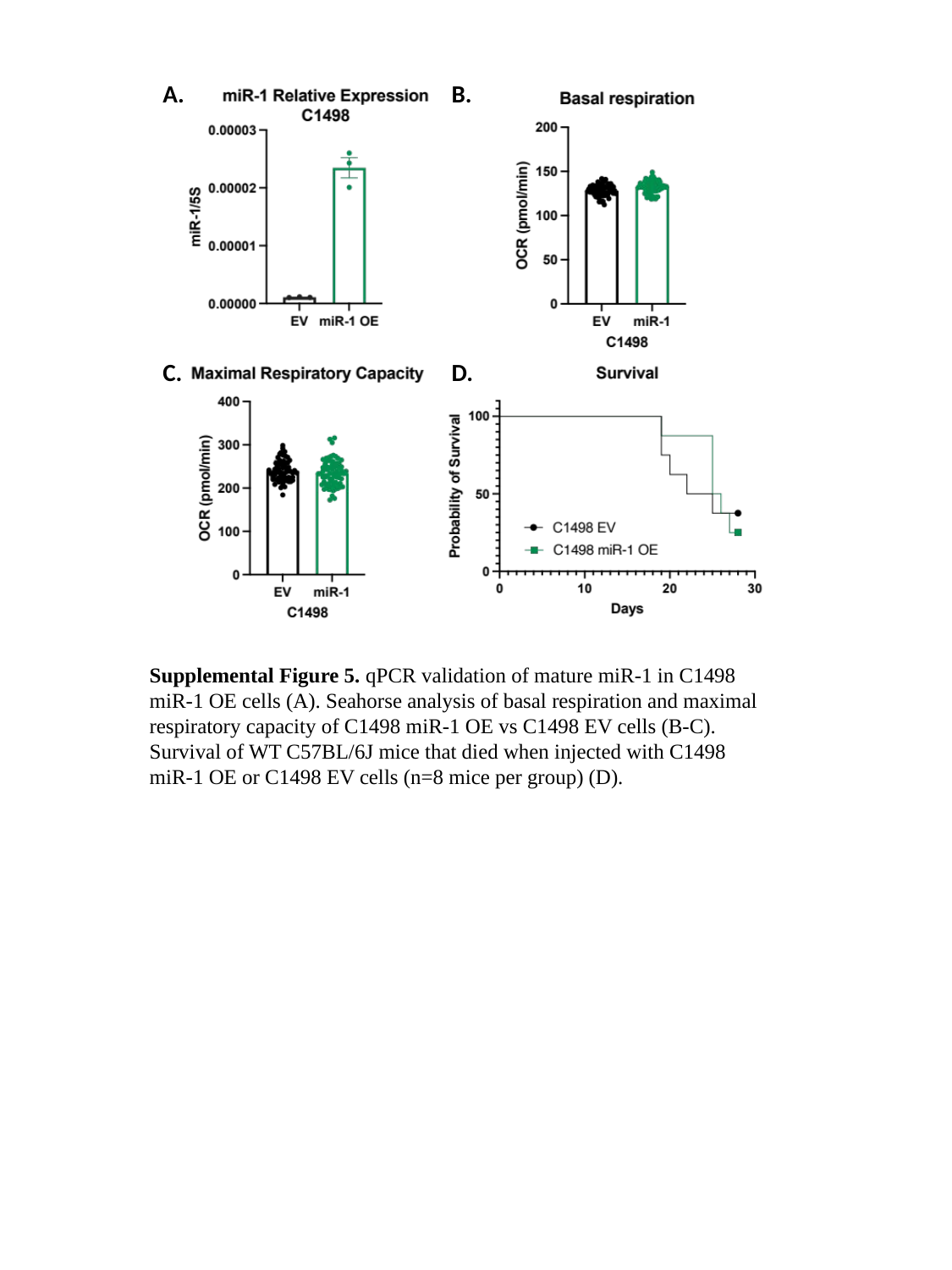

A.
B.
D.
C.
Supplemental Figure 5. qPCR validation of mature miR-1 in C1498 miR-1 OE cells (A). Seahorse analysis of basal respiration and maximal respiratory capacity of C1498 miR-1 OE vs C1498 EV cells (B-C). Survival of WT C57BL/6J mice that died when injected with C1498 miR-1 OE or C1498 EV cells (n=8 mice per group) (D).
