# Supplementary figures and images for: miRNA-1 promotes acute myeloid leukemia cell pathogenesis through metabolic regulation
Source: Front Genet. 2023 May 9;14:1192799. doi: 10.3389/fgene.2023.1192799 (PMC10203238; doi:10.3389/fgene.2023.1192799)

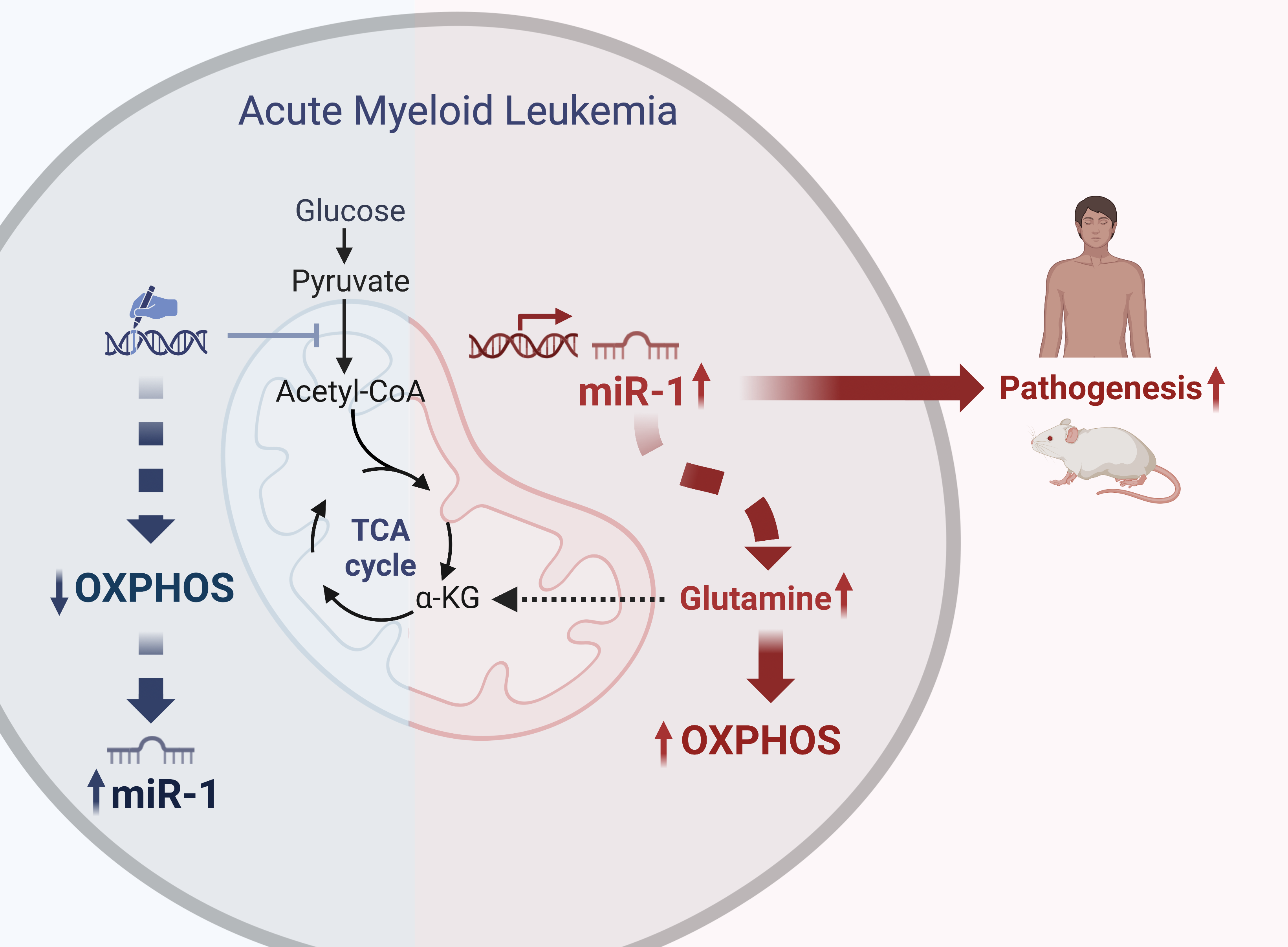

Supplement: Supplementary file 2 [file Image1.PNG]
