# Supplementary material for: miRNA-1 promotes acute myeloid leukemia cell pathogenesis through metabolic regulation
Source: Front Genet. 2023 May 9;14:1192799. doi: 10.3389/fgene.2023.1192799 (PMC10203238; doi:10.3389/fgene.2023.1192799)
Supplement: Supplementary file 3 [file DataSheet1.DOCX]

***Supplementary material***

**miR-1 promotes Acute Myeloid Leukemia cell pathogenesis through metabolic regulation**

Arevik Ghazaryan^1^, Jared A. Wallace^1^, William W. Tang^1^, Cindy Barba^1^, Soh-Hyun Lee^1^, Kaylyn M. Bauer^1^, Morgan C. Nelson^1^, Carissa Kim^1^, Chris Stubben^2^, Warren P. Voth^1^, Dinesh S. Rao^3,^ Ryan M. O’Connell^1 2^*

*Corresponding author. Email: ryan.oconnell@path.utah.edu (R.M.O.)

**Extensive description of Materials and Methods**

**Cell culture and cell lines**

MV4-11, Molm-14, C1498,THP-1 cell lines were purchased from ATCC and cultured in comlete RPMI media ((10% FBS, L-glutamine, β-Mercaptoethanol )BME), Pen/Strep antibiotics, HEPES, Sodium Pyruvate, and Non-essential amino acids). The cells were grown in normoxic conditions, with 5% CO2 at 37C.

MPC1-KO MV4-11, Molm-14, THP-1 cells were generated using previously described CRISPR/Cas9(1) lentiCRISPRv2 construct containing a GFP selection marker and one specific short guide RNA sequence against human *MPC1*.

miR-1-3p overexpressing MV4-11 and Molm-14 cell lines were created using ABM pLenti-EF1a-GFP promoter with GFP followed by Origene (CAT#: SC400037) insert that produces GFP and mature UGGAAUGUAAAGAAGUAUGUAU miR-1-2 (miR-1-2 OE) or just GFP (empty vector (EV)).

We overexpressed miR-1-3p in C1498 cell lines by cloning MSCV into ABM pLenti-EF1a-GFP promoter, and replacing pLenti-EF1a-GFP promoter with MSCV -EF1a-GFP promoter, with GFP followed by Origene (CAT#: SC400037) insert that produces GFP and mature UGGAAUGUAAAGAAGUAUGUAU miR-1-2 (miR-1-2 OE) or just GFP (empty vector (EV)).

**Mice**

Mice used for xenograft study are NSG NOD-*scid* IL2Rgamma^null^, gift from Alana Welm lab. MV4-11 EV or MV4-11 miR-1 OE cells were washed twice with PBS and 1.3 mln cells in 200ul PBS were injected through tail vain twice in two consecutive days. For C1498 mouse AML experiment C57BL/6J mice were purchased from The Jackson Laboratory (Strain #:000664). C1498 EV or C1498 miR-1 OE cells were washed twice with PBS and 2.5 mln cells in 200ul PBS were injected through tail. Mice were housed in a vivarium with a 12-hour light/dark cycle per day, temperatures of 22°C and 20% relative humidity. All experimental procedures and husbandry were performed with the approval of the Institutional Animal Care and Use Committee and the Comparative Medicine Center of the University of Utah. Littermates and male mice were used for all experiments and mice were 8-12 weeks old at the beginning of experiments.

**qRT-PCR**

RNA was isolated by following manufacturer protocols using either the Qiagen miRNeasy Mini. cDNA was generated according to manufacturer’s protocols with qSCRIPT cDNA SuperMix (QuantaBio) for mRNA (using 100 ng RNA input) and miRNA LNA RT kit (Qiagen) for miRNA reactions (100 ng RNA input). hsa-miR-1-3p primer was purchased from Qiagen (YP00204344). All reactions were run on an Applied Biosciences QS6 Thermocycler.

**Metabolomics**

7.9 million MV4-11 miR-1 or MV4-11 EV cells (n=6) were used for analysis. Cold 90% methanol (MeOH) solution was added to cell pellet to give a final concentration of 80% MeOH. Samples were then incubated at -20 ˚C for 1 hr. After incubation the samples were centrifuged at 20,000 x g for 10 minutes at 4 ˚C. The supernatant was then transferred from each sample tube into a labeled, fresh micro centrifuge tube. Pooled quality control samples were made by removing a fraction of collected supernatant from each sample and process blanks were made using only extraction solvent and no cell culture. The samples were then dried en vacuo. All GC-MS analysis was performed with an Agilent 5977b GC-MS MSD-HES fit with an Agilent 7693A automatic liquid sampler. Data was collected using MassHunter software (Agilent). Metabolites were identified and their peak area was recorded using MassHunter Quant.

**miRNA-mRNA sequencing/bioinformatic analysis**

mRNA was isolated by following manufacturer protocols using the Qiagen miRNeasy Mini kit (Cat.: 217084). The cell ectracts were treated with Qiagen RNase free DNase by following manufacturer protocol (Cat.: 79254). The human GRCh38 genome and gene annotation files were downloaded from Ensembl release 102 and a reference database was created using STAR version 2.7.6a with splice junctions optimized for 150 base pair reads(2). The trimmed reads were aligned to the reference database using STAR in two pass mode to output a BAM file sorted by coordinates. Mapped reads were assigned to annotated genes using featureCounts version 1.6.3(3). The output files were summarized using MultiQC to check for any sample outliers(4). Differentially expressed genes were identified using a 5% false discovery rate with DESeq2 version 1.30.0(5).

**TCGA analysis**

The clinical and miRNA expression data from TCGA LAML were downloaded from the Genomic Data Commons (GDC) using the TCGAbiolinks package(6). The raw counts from 181 patients were analyzed using DESeq2 version 1.30.0. A survival table was created from the clinical data with the number of days to death or the days to the last follow up. The log2 normalized counts from significant genes were analyzed using a proportional hazards regression model in R (coxph function). Survival curves were plotted using high and low expression groups (50th percentiles). The FLT3-ITD+ AML miRNA expression profile was taken from our previous analysis(7). The results shown here are in whole or part based on data generated by the TCGA Research Network (http://cancergenome.nih.gov/). The content is solely the responsibility of the authors and does not necessarily represent the official views of the National Cancer Institute or the NIH.

The clinical and miRNA expression data from TARGET-AML were downloaded from the Genomic Data Commons (GDC) using the TCGAbiolinks package. The raw counts from 60 patients with IDT status were analyzed using DESeq2 version 1.30.0. Differentially expressed miRNAs in IDT positive vs. IDT negative were identified using a 5% false discovery rate while controlling for any sex-related effects.

**Western Blot**

Cell Signaling MPC1 (D2L9I) Rabbit mAb #14462 antibody was used for MPC1 Western blots to confirm MPC-1 Protein reduction. The western blotting was performed based on manufacturer’s protocol for MPC1. GAPDH Santa Cruz Antibody (0411): sc-47724 was used as control.

**Cell Proliferation**

BrdU Cell Proliferation Assay Kit #6813 was used to measure cell proliferation. 10,000 MV4-11 EV or miR-1 OE cells were used to plate the 96 well plate for the assay. The assay was performed based on manufacturer’s protocol.

**Cell death and Apoptosis Flow Analysis**

To meausure cell death we stained 500.000 MV4-11 EV or miR-1 OE cells were washed and stained with 7AAD (2 µL/test)(7-AAD Viability Staining Solution, BioLegend) and Annexin V (2 µL/test)(Pacific Blue; BioLegend) in Annexin binding buffer (BioLegend; cat # 422201) for 20 min in room temperature. The flow cytometry was performed using a BD LSRFortessa II (BD Biosciences).

**Mitochondrial and Cellular Assays**

1 million MV4-11 EV or miR-1 OE cells were plated in 96 well plates. The cells were washed with cold PBS 2X and stained either with Fisher Scientific MitoSOX Red (M36008)(1:10000 in Hank's Balanced Salt Solution (HBSS) without Mg and Ca) to meausure mitochondrial superoxide, or with Fisher Scientific ThiolTracker™ Violet (T10095)(1:1000 in HBSS without Mg and Ca) to meausure cellular glutathione. The flow analysis was used for data collection (BD LSRFortessa II (BD Biosciences)).

For Mitotracker analysis 100,000 cells were seeded in 96 well plates and the cells were stained in complete 10% FBS RPMI Media with MitoTracker™ Red Invitrogen™ M22425 (1:1000) and incubated in for 20 minute in 5% CO2 at 37C.

**Primers used in this study:**

| **Gene** | **Forward (5’-3’)** | **Reverse (5’-3’)** |
| --- | --- | --- |
| **hs *MPC1*** | GTGCGGAAAGCGGCGGACTA | GGCAGCAATGGGAAGACCCCA |
| **hs *MPC1*** | CGCGTTGGTGCGGAAAGCG | GGCAAATGTCATCCGCCCACTGA |
| **hs L32** | AGCTCCCAAAAATAGACGCAC | TTCATAGCAGTAGGCACAAAGG |

**Supplemental Figure Legends**

**Supplemental Figure 1.** Western Blot of Molm-14 MPC-1 protein in EV and MPC-1 KO cells (A). Seahorse assay OCR and ECAR plots for Molm-14 (B-C) and MV4-11 (D-E) EV or MPC-1 KO cells. miR-1-3p expression with normalized counts for MV4-11 and Molm-14 EV vs MPC-1 Ko cells (F). qPCR confirmation of MPC1 gene deletion by CRISPR/Cas9 construct in THP-1 cells (G). miR-1 expression of THP-1 MPC1 KO vs WT cells (H). Unpaired two-tailed t test. **** p = <0.0001.

**Supplemental Figure 2.** Patients from TCGA dataset were divided into 2 groups on the 75th percentile high and 25th percentile low (A-B) based on their mature miR-1-3p expression, and 50th percentile high and 50th percentile low (C-D). Gehan-Breslow-Wilcoxon test * p = < 0.05, ** p = < 0.01 was used for survival plots.

**Supplemental Figure 3.** Diagram of miR-1 overexpression pLenti III EF1a GFP Hs MIR1-2 (OE) or empty vector pLenti-III-mir-GFP Control Vector (EV) expressing lentivectors (A). Diagram of luciferase PGK1 - Oplophorus (NanoLuc) Luciferase Reporter (Luc) and miR-1 binding sites PGK1 - Oplophorus (NanoLuc)-miR1-3p Sensor Luciferase Reporter (BS) expressing vectors (B). Volcano plot of statistically significantly altered genes in miR-1 OE MV4-11 (C) and Molm-14 (D) cells compared to EV expressing MV4-11 and Molm-14 control cells respectively with p = <0.05.

**Supplemental Figure 4.** Seahorse assay OCR plots for MV4-11 (A) and Molm-14 (B) EV or miR-1 OE cells. Cell proliferation (C), cell death (D) and apoptosis (E) for MV4-11 EV and miR-1 OE cells. MFI of flow analysis for Mitosox (F), Mitotracker (G) and Thioltracker (H) assays for MV4-11 EV and miR-1 OE cells.

**Supplemental Figure 5.** qPCR validation of mature miR-1 in C1498 miR-1 OE cells (A). Seahorse analysis of basal respiration and maximal respiratory capacity of C1498 miR-1 OE vs C1498 EV cells (B-C). Survival plots of WT C57BL/6J mice when injected with C1498 miR-1 OE or C1498 EV cells (n=8 mice per group) (D).

**References**

1. Ramstead AG, Wallace JA, Lee SH, Bauer KM, Tang WW, Ekiz HA, et al. Mitochondrial Pyruvate Carrier 1 Promotes Peripheral T Cell Homeostasis through Metabolic Regulation of Thymic Development. Cell Rep. 2020;30(9):2889-99 e6.

2. Dobin A, Davis CA, Schlesinger F, Drenkow J, Zaleski C, Jha S, et al. STAR: ultrafast universal RNA-seq aligner. Bioinformatics. 2013;29(1):15-21.

3. Liao Y, Smyth GK, Shi W. featureCounts: an efficient general purpose program for assigning sequence reads to genomic features. Bioinformatics. 2014;30(7):923-30.

4. Ewels P, Magnusson M, Lundin S, Kaller M. MultiQC: summarize analysis results for multiple tools and samples in a single report. Bioinformatics. 2016;32(19):3047-8.

5. Love MI, Huber W, Anders S. Moderated estimation of fold change and dispersion for RNA-seq data with DESeq2. Genome Biol. 2014;15(12):550.

6. Colaprico A, Silva TC, Olsen C, Garofano L, Cava C, Garolini D, et al. TCGAbiolinks: an R/Bioconductor package for integrative analysis of TCGA data. Nucleic Acids Res. 2016;44(8):e71.

7. Wallace JA, Kagele DA, Eiring AM, Kim CN, Hu R, Runtsch MC, et al. miR-155 promotes FLT3-ITD-induced myeloproliferative disease through inhibition of the interferon response. Blood. 2017;129(23):3074-86.
